# Non-invasive estimation of split renal function from routine ^68^Ga-SSR-PET/CT scans

**DOI:** 10.3389/fmed.2023.1169451

**Published:** 2023-06-28

**Authors:** Matthias Weissinger, Kyra Celine Seyfried, Stephan Ursprung, Salvador Castaneda-Vega, Ferdinand Seith, Sebastian von Beschwitz, Jonas Vogel, Patrick Ghibes, Konstantin Nikolaou, Christian la Fougère, Helmut Dittmann

**Affiliations:** ^1^Department of Diagnostic and Interventional Radiology, University Hospital Tuebingen, Tuebingen, Germany; ^2^Department of Nuclear Medicine and Clinical Molecular Imaging, University Hospital Tuebingen, Tuebingen, Germany; ^3^Department of Preclinical Imaging and Radiopharmacy, Werner Siemens Imaging Center, Eberhard Karls University Tuebingen, Tuebingen, Germany; ^4^iFIT-Cluster of Excellence, Eberhard Karls University Tuebingen, Tuebingen, Germany; ^5^German Cancer Consortium (DKTK), Partner Site Tuebingen, Tuebingen, Germany

**Keywords:** accumulation index, DOTATATE, NET (neuro-endocrinal tumors), PRRT (peptide receptor radionuclide therapy), split renal function, SSR PET/CT, single-sided renal function, total kidney accumulation

## Abstract

**Objective:**

Patients with impaired kidney function are at elevated risk for nephrotoxicity and hematotoxicity from peptide receptor radionuclide therapy (PPRT) for advanced neuroendocrine tumors. Somatostatin receptor (SSR)-PET/CT imaging is the method of choice to identify sufficient SSR expression as a prerequisite for PRRT. Therefore, our study aimed to explore whether split renal function could be evaluated using imaging data from routine SSR-PET/CT prior to PRRT.

**Methods:**

In total, 25 consecutive patients who underwent SSR-PET/CT (Siemens Biograph mCT^®^) before PRRT between June 2019 and December 2020 were enrolled in this retrospective study. PET acquisition in the caudocranial direction started at 20 ± 0.5 min after an i.v. injection of 173 ± 20 MBq [^68^Ga]Ga-ha DOTATATE, and the kidneys were scanned at 32 ± 0.5 min p.i. The renal parenchyma was segmented semi-automatically using an SUV-based isocontour (SUV between 5 and 15). Multiple parameters including SUVmean of renal parenchyma and blood pool, as well as parenchyma volume, were extracted, and accumulation index (**ACI**: renal parenchyma volume/SUVmean) and total kidney accumulation (**TKA**: SUVmean x renal parenchyma volume) were calculated. All data were correlated with the reference standard tubular extraction rate (TER-MAG) from [^99m^Tc]Tc-MAG3 scintigraphy and glomerular filtration rate (GFR_CDK − EPI_).

**Results:**

SUVmean of the parenchymal tracer retention showed a negative correlation with TER_MAG_ (*r*: −0.519, *p* < 0.001) and GFR_CDK − EPI_ (*r*: −0.555, *p* < 0.001) at 32 min p.i. The herein-introduced ACI revealed a significant correlation (*p* < 0.05) with the total tubular function (*r*: 0.482), glomerular renal function (*r*: 0.461), split renal function (*r*: 0.916), and absolute single-sided renal function (*r*: 0.549). The mean difference between the split renal function determined by renal scintigraphy and ACI was 1.8 ± 4.2 % points.

**Conclusion:**

This pilot study indicates that static [^68^Ga]Ga-ha DOTATATE PET-scans at 32 min p.i. may be used to estimate both split renal function and absolute renal function using the herein proposed “Accumulation Index” (ACI).

## 1. Introduction

Peptide receptor radionuclide therapy (PRRT) with the lutetium 177-labeled sandostatin analog (^177^Lu)-oxodotreotide (e.g., Lutathera^®^) is a well-tolerated and approved second-line therapy for the treatment of advanced midgut neuroendocrine tumors (1, 2). PRRT is administered in combination with nephroprotective amino acids in multiple cycles usually 6–12 weeks apart (1, 2).

The kidneys are exposed to a high radiation dose in PRRT. The theranostic somatostatin analog is predominantly glomerular filtrated and actively reabsorbed in the proximal renal tubules by the endocytic megalin receptor (3, 4). This results in a high radiation dose to the renal cortex (3). Based on the traditional dose limit established for external beam irradiation and ^177^Lu and ^90^Y PRRT dosimetry, the absorbed dose of kidneys should be restricted to 23–40 Gy (depending on risk factors) (5), which is often exceeded when multiple cycles of PRRT are applied in routine clinical practice (1, 2, 6). Early PRRT using 90-Yttrium-labeled somatostatin analogs reported severe nephrotoxicity grade 3/4 in approximately 3% of all patients (7, 8). PRRT with (^177^Lu)-oxodotreotide has shown to be considerably less nephrotoxic (7). However, up to 25% of all patients treated with ^177^Lu PRRT develop a mild renal toxicity grade 1/2, which was persistent in about half of the patients (1, 7, 9). Nephrotoxicity was not only associated with previous 90-Yttrium PRRTs and the number of PRRT cycles but also with hypertension and other nephrotoxic risk factors (7).

Notably, preexisting poor renal function has been identified as a risk factor for deteriorated kidney function after PRRT (7, 8, 10, 11). Reduced renal function results in higher radiation doses to the kidneys and increases the risk of hematotoxicity (10). Thus, it can be assumed that unilateral renal insufficiency will result in disproportionate ipsilateral radiation damage during PRRT. Unilateral renal dysfunction that is partially compensated by the contralateral kidney remains undetected in laboratory testing and can be diagnosed, at present, only by imaging techniques. Renal scintigraphy is the gold standard for determining split renal function (12). Renal scintigraphy with ^99m^Technetium-mercaptoacetyltriglycine (MAG3) was introduced in 1984 and became the method of choice for the precise determination of the split renal function, tubular function, and renal plasma flow, and defines the gold standard in most clinical trials (12, 13). However, renal function scintigraphy is an additional examination for the patient, associated with additional time spent in the hospital and cost.

An interesting approach to avoid additional time-consuming or potentially harmful tests is the integration of split renal function estimation into other routine imaging procedures. For instance, Geist et al. described a method to determine side-separated glomerular filtration rate (GFR) and renal plasma flow using a short dynamic FDG PET/MRI imaging protocol (14). These short dynamic sequences can be acquired within the setting of a clinical PET scan without the need for additional tracer, contrast media, or radiation exposure and might save the patient an extra hospital appointment.

Integrating renal function tests into the PET workflow is clinically very attractive, especially for patients requiring periodic PET/CT follow-up and regular renal function monitoring due to potentially nephrotoxic therapy such as PRRT.

Therefore, this study aimed to determine split renal function using data from routine static somatostatin receptor (SSR)-PET/CT with the predominately glomerular filtrated [^68^Ga]Ga-ha DOTATATE tracer and to correlate results with renal scintigraphy and serum GFR as reference standards.

## 2. Materials and methods

This retrospective study enrolled patients with predominantly non-resectable and/or metastasized neuroendocrine tumors who underwent [^68^Ga]Ga-ha DOTATATE PET/CT and [^99m^Tc]Tc-mercaptoacetyltriglycine (MAG3) renal scintigraphy before PRRT between June 2019 and December 2020. Patients with a history of potentially nephrotoxic chemotherapy or PRRT within 5 years before PET/CT were excluded from the analysis. All patients agreed to the anonymized data analysis and provided written informed consent to the scientific analysis of pseudonymized medical data collected at our University Hospital. The institutional review board approved the retrospective data analyses (registry No. 386/2021BO2). One patient underwent an additional dynamic PET acquisition for tracer kinetic measurements within a review board approved prospective dynamic PET/CT imaging trial (registry No. 863/2019BO1, DRKS 00021217).

### 2.1. [^68^Ga]Ga-ha DOTATATE PET/CT

Whole-body [^68^Ga]Ga-ha DOTATATE PET/CT comprising skull to mid-thigh was performed on a Biograph mCT^®^ scanner, (Siemens Healthineers) with continuous-bed-motion technique (Siemens FlowMotion^®^). PET acquisition started 20 ± 0.5 min after an i.v. injection of 173 ± 20 MBq ^68^Ga-ha-DOTATATE in caudocranial direction. No diuretics were administered. The kidneys were imaged by PET 32 ± 0.5 min p.i. Weight and size for mean standardized uptake value (SUVmean) calculation were measured for each patient immediately before the PET/CT examination. Patients were asked to drink 1,000 ml of oral contrast media solution (1,000 ml of mannitol 2.5%) starting 1 h before tracer application. Patients were positioned on the PET/CT bed on a vacuum mattress and asked to breathe as shallowly and evenly as possible and were asked not to move. The PET/CT examination started with a contrast-enhanced diagnostic multi-slice CT (80–120 ml Ultravist 370^®^ i.v., arterial and portal-venous phase) except for contraindications. PET imaging data were reconstructed by applying an iterative OSEM 3D algorithm (256^*^256 matrix) with a 4 mm Gaussian filter.

### 2.2. Renal scintigraphy

All patients were asked to drink at least 10 ml of mineral water/kg body weight 30 min before scintigraphy. Each individual underwent dynamic renal scintigraphy starting with a bolus injection of 100 ± 6 MBq [^99m^Tc]Tc-MAG3 on a SPECT or SPECT/CT camera. Camera heads were in H-mode, and the acquisition was done in a dorsal view. Regions of interest (ROI) were placed in the renal parenchyma including the renal pelvic caliceal system (RPCS) and aorta by technicians with more than 5 years of experience in renal scintigraphy and adjusted in case of motion artifacts. Calculation of split renal function was obtained by the activity increase over 60 s starting 45–60 s p.i. The tubular extraction rate of [^99m^Tc]Tc-MAG3 was quantified using Bubeck's method (15) from single blood samples 20 and 30 min p.i. Side-separated TER-MAG was calculated by multiplying the split renal function with TER. All estimates were validated by a nuclear medicine specialist.

### 2.3. Glomerular function rate calculation

The glomerular function rate was calculated from serum creatinine levels using the clinically established CKD-EPI formula (16). Serum creatinine was measured on the day of renal scintigraphy, as well as a maximum of 2 weeks before PET/CT examination.

### 2.4. Semiquantitative PET/CT measurements and renal parenchyma segmentation

Segmentation of renal parenchyma and RPCS in PET/CT images was performed with a HERMES SMART^®^ Workstation v2.13.0.37 (HERMES MEDICAL SOLUTIONS) by a PhD student (K.S.) and validated by an expert in hybrid imaging (M.W). SUVmean of the renal parenchyma and the parenchymal volume were quantified for each kidney by the following methods.

First, a freehand volume of interest (VOI) was placed over the kidney excluding the renal pelvis in the fused PET/CT images. Overlaps with other organs, such as the liver or adrenal glands, were strictly avoided. Second, an isocontour was applied to the VOI. The volume of [^68^Ga]Ga-ha DOTATATE avid renal parenchyma was segmented semi-automatically using a lower threshold of SUV = 5.0, which has shown the best visual fit to the anatomy, and an upper threshold of SUV = 15 was used to exclude radioactive urine extracted in the RPCS. In addition, the following indexes were calculated.

**Total Kidney Accumulation:**


$$\begin{array}{l} TKA_{\text{5}-\text{15}}={\text{SUV mean}}_{\text{5}-\text{15 isocontour}}\times [\text{68Ga}]\text{Ga }\\ \;-\text{ha DOTATATE avid renal parenchyma} \end{array}$$

**Total Kidney Accumulation with blood pool correction:**


$$\begin{array}{l} {}_{\text{bpc}}{\text{TKA}}_{5-15}=({\text{SUV mean}}_{\text{5}-\text{15 isocontour}}-\text{SUV mean blood pool})\;\\ \;\times \;{[}^{\text{68}}\text{Ga}]\text{Ga-ha DOTATATE avid renal parenchyma} \end{array}$$

**Accumulation Index:**


$$ACI_{5-15}=\frac{\;[\text{68Ga}]\text{Ga}-\text{ha DOTATATE avid renal parenchyma}}{\text{SUV mean }(\text{5}-\text{15 isocontour})}$$

For the left kidney, split renal function is expressed as a percentage.


$$\begin{array}{l} \text{Split renal function}=\frac{left\;kidney}{left+right\;kidney}\times \;100 \end{array}$$

The activity concentration of [^68^Ga]Ga-ha DOTATATE in the blood pool was measured semiquantitatively per SUVmean within a cylindric VOI placed in the descending aorta with a volume of ≥5 ml.

### 2.5. Tumor segmentation

Freehand 3D VOI was plotted around the tumor areas by an expert in hybrid imaging (M.W.) using Hermes Affinity v.3.0.5 (Hermes Medical Solutions). The tumor segmentation was PET based using an isocontour with an individual threshold defined over the SUVmax of the adjacent healthy tissue. A tissue density of 1 g/ml was assumed for the calculation of the fraction of the injected dose. The following parameters were obtained:

**Metabolic Volume:**


$$\begin{array}{l} \text{MTV}=\text{SUVmean }\times \text{ VOI of segmented tumor} \end{array}$$

**Fraction of the injected dose:**


$$\begin{array}{l} \text{\%ID}=\frac{\text{MTV}}{\text{body weight }\times \text{ SUV }1} \end{array}$$

### 2.6. Statistical analysis

Renal scintigraphy is defined as the gold standard for absolute and side-separated tubular renal function. Statistical analysis was performed using SPSS Statistics v.28.0 (IBM Inc.) and Excel^®^ 2019 v.1808 software (Microsoft).

Significance testing was performed for interval-scaled data using the Students' *t*-test. A *p*-value of <0.05 was considered statistically significant. A Pearson's correlation coefficient of *r* >0.7 was defined as strong, 0.7–0.3 as moderate, and <0.3 as a weak linear correlation.

## 3. Results

### 3.1. Patients' characteristics

In total, 25 patients met the inclusion criteria. All patients suffered from metastatic tumors, with predominantly neuroendocrine differentiation (details in Table 1) and underwent SSR-PET/CT for the evaluation of PRRT. Gender was balanced with 13 male and 12 female patients. The average patient age on the day of the PET/CT examination was 64.4 ± 15.6 years (range: 21–81 years) with average patient size and weight of 166.4 ± 19 cm and 69.3 ± 20.3 kg. Male patients were non-significantly older, larger, and heavier than female patients at the timepoint of PET/CT (*p* = 0.290–0.558). The mean GFR was 79.4 ± 20.9 ml/min/1.73 m^2^. In total, 4 out of the 25 patients presented with moderately impaired kidney function and chronic kidney disease (CKD) stage 3 (3a: *n* = 3, 3b: *n* = 1), and 14 out of the 25 patients had mild impaired kidney function, CKD Stage 2. The average time between renal scintigraphy and PET/CT was 15.9 ± 40.6 days and was similar between genders in the two-sided *t*-test (*p* = 0.162).

**Table 1:** Detailed listing of the participants' underlying disease and prior therapies.

**Patient ID**	**Sex**	**Age in years**	**Primary Tumor**	**Tumor Stage**	**Proliferation index Ki 67**	**Prior Therapy**	**GFR ml/min/1.73m^2^**	**MTV/%ID**
1	Male	74	Ileum NET	T3, N1, M0	< 10%	Surgery	66	161/0.2%
2	Female	67	Ileum NET	Tx, Nx, M1	17.8%	Surgery, SSA, PRRT	78	1,371/1.8%
3	Male	72	Atypical pulmonary carcinoid	T4, Nx, M1	20%	SSA	64	3,077/3.4%
4	Female	78	Parotid Gland NET	Tx, Nx, M1	20%	Surgery, RT	40	104/0.1%
5	Female	54	Pancreas NET	T4, Nx, M1	3–5%	SSA	95	1,815/2.6%
6	Female	68	CUP NET	Tx, N1, M1	15%	SSA	100	1,899/27.2%
7	Male	68	Ileum NET	T3, N1, M1	5%	Surgery, SSA	54	1,121/1.4%
8	Female	63	Duodenum NET	Tx, Nx, M1	2–5%	None	77	246/0.5%
9	Male	71	Pancreas NET	Tx, Nx, M1	5%	SSA	67	1,686/20.8%
10	Female	79	Pancreas NET	T3, N1, M1	5%	Surgery	69	732/2.1%
11	Male	24	Pancreas NET	Tx, Nx, M1	20%	SSA	98	1,041/1.6%
12	Female	51	Pancreas NET	Tx, Nx, M1	40%	None	89	14,002/24.4%
13	Male	79	CUP NET	Tx, N1, M1	< 1%	SSA	83	12,448/18.6%
14	Male	60	Ileum NET	T3, N1, M1	< 2%	Surgery, SSA	100	8/0.1 %
15	Female	65	Ileum NET	Tx, N1, M1	-	Surgery, PRRT, SSA	68	342/0.6%
16	Male	70	Ileum NET	T4, N1, M1	< 2%	Surgery, SSA	95	8,574/10.2%
17	Male	79	Ileum NET	T3, N1, M1	3%	Surgery, SSA	85	809/1.0%
18	Male	61	Oncocytic thyroid carcinoma	T3, N0, M1	-	Surgery, RT, RIT	70	2,475/2.2%
19	Female	55	Ileum NET	T3, N1, M1	5–7 %	Surgery, SSA	59	873/1.1%
20	Male	81	Pancreas NET	Tx, Nx, Mx	5–10%	SSA	85	1,691/2.8%
21	Male	51	Pancreas NET	Tx, Nx, M1	25–30%	None	109	17,228/25.5%
22	Female	65	CUP NET	Tx, Nx, M1	5%	Surgery	66	1,836/2.9%
23	Male	78	Ileum NET	Tx, N1, M1	< 1%	Surgery, SSR, CT	59	2,764/4.6%
24	Female	21	Pancreas NET	T3, N1, M1	5 %	Surgery, CT	139	550/0.9%
25	Female	77	CUP NET	Tx, Nx, M1	25%	Surgery	70	280/0.5%

CT, Chemotherapy; NET, Neuroendocrine Tumor; PRRT, Peptide Receptor Radionuclide Therapy; RIT, Radioiodine Therapy RT, Radiotherapy; SSA, Somatostatin Analog therapy.

### 3.2. Tracer kinetics

**Figure 1:**
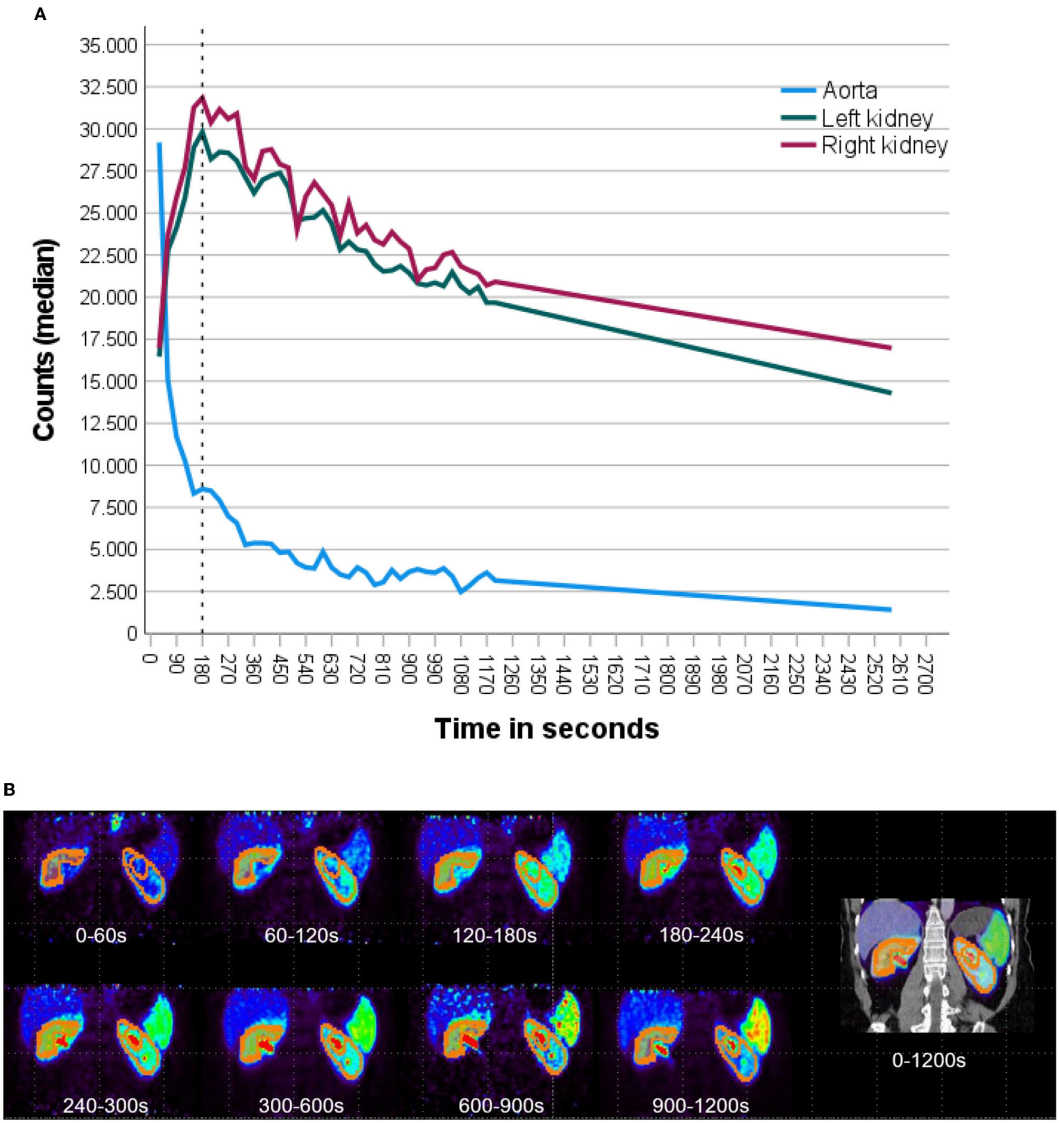
Dynamic SSR-PET acquisition of a patient with an initial diagnosis of NET with normal renal function and without previous nephrotoxic therapies was analyzed. **(A)** Time–activity curve was plotted from dynamic [^68^Ga]Ga-ha DOTATATE PET 0–20 min (30 s frames) after i.v. tracer injection followed by static acquisition at 43 min for the left (green) and right (purple) kidney. [^68^Ga]Ga-ha DOTATATE concentration reached its peak in the renal parenchyma 180 s after tracer injection followed by a continuous decrease until the end of the examination 43 min p.i. Blood pool activity measured in the abdominal aorta presents with a rapid exponential decrease. **(B)** PET images of [^68^Ga]Ga-ha DOTATATE tracer distribution at different time points p.i. with plotted isocontours of semiautomatic parenchyma segmentation. Renal parenchyma demarcates clearly from the background and adjacent organs at all time points. The isocontour used for renal parenchymal volumetry removes background (SUV <5) and excreted urine in the pelvicocaliceal system (SUV >15).

As a proof of concept, [^68^Ga]Ga-ha DOTATATE kinetics in the renal parenchyma was measured using a dynamic PET acquisition in one patient without renal function impairment during oncological staging. [^68^Ga]Ga-ha DOTATATE concentration reached its peak in the renal parenchyma 180 s after bolus injection followed by an exponential decrease until the end of the examination 43 min p.i., as shown in Figure 1.

### 3.3. Total renal function

**Figure 2:**
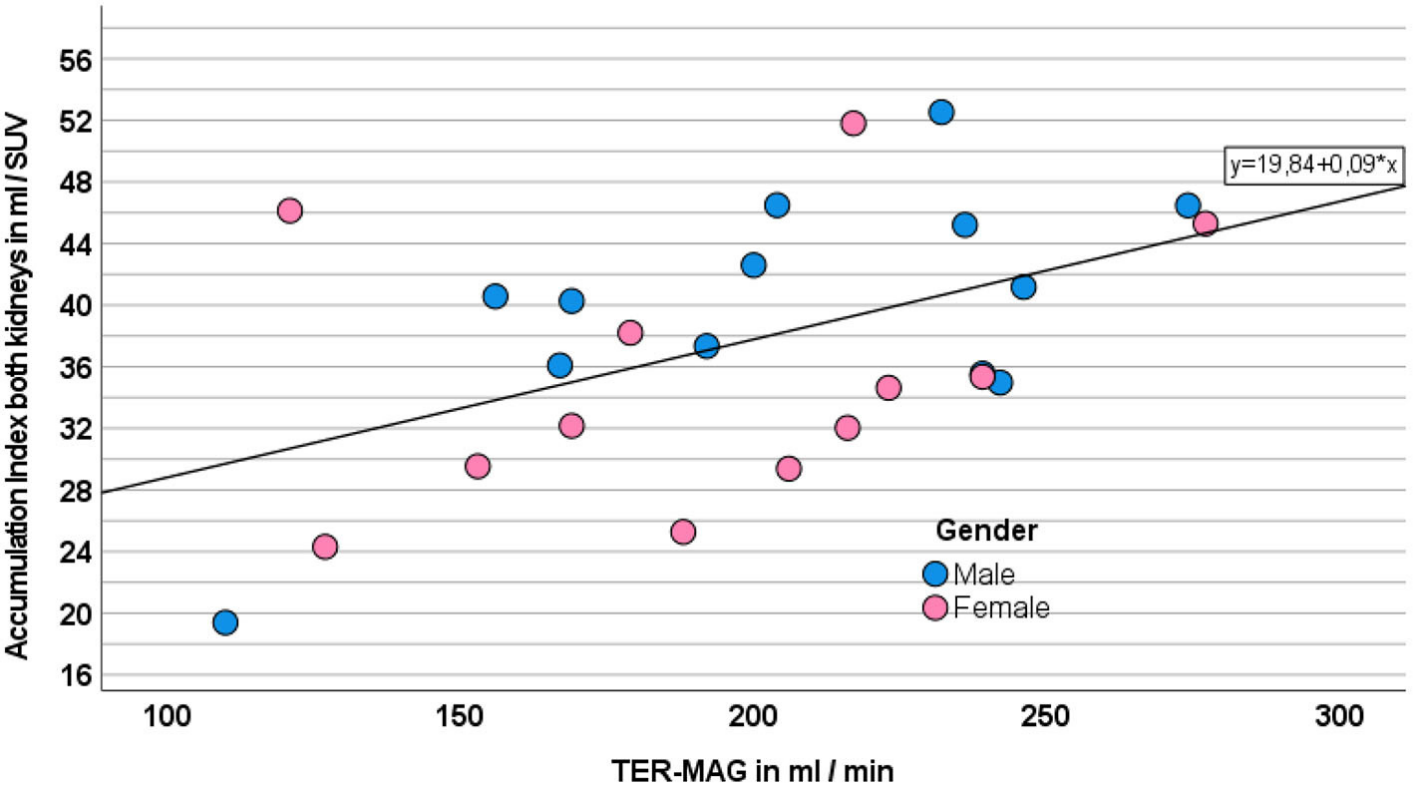
Correlation between ACI as the sum of both kidneys and TER-MAG. Gender is displayed in different colors.

TER-MAG revealed a strong correlation with GFR_CDK − EPI_ (*r*: 0.756, *p* < 0.001). The calculated accumulation index (ACI) showed a moderate correlation with TER-MAG (*r*: 0.482, *p* = 0.015, Figure 1) and GFR_CDK − EPI_ (0.461, *p* = 0.020) as shown in Figure 2.

**Figure 3:**
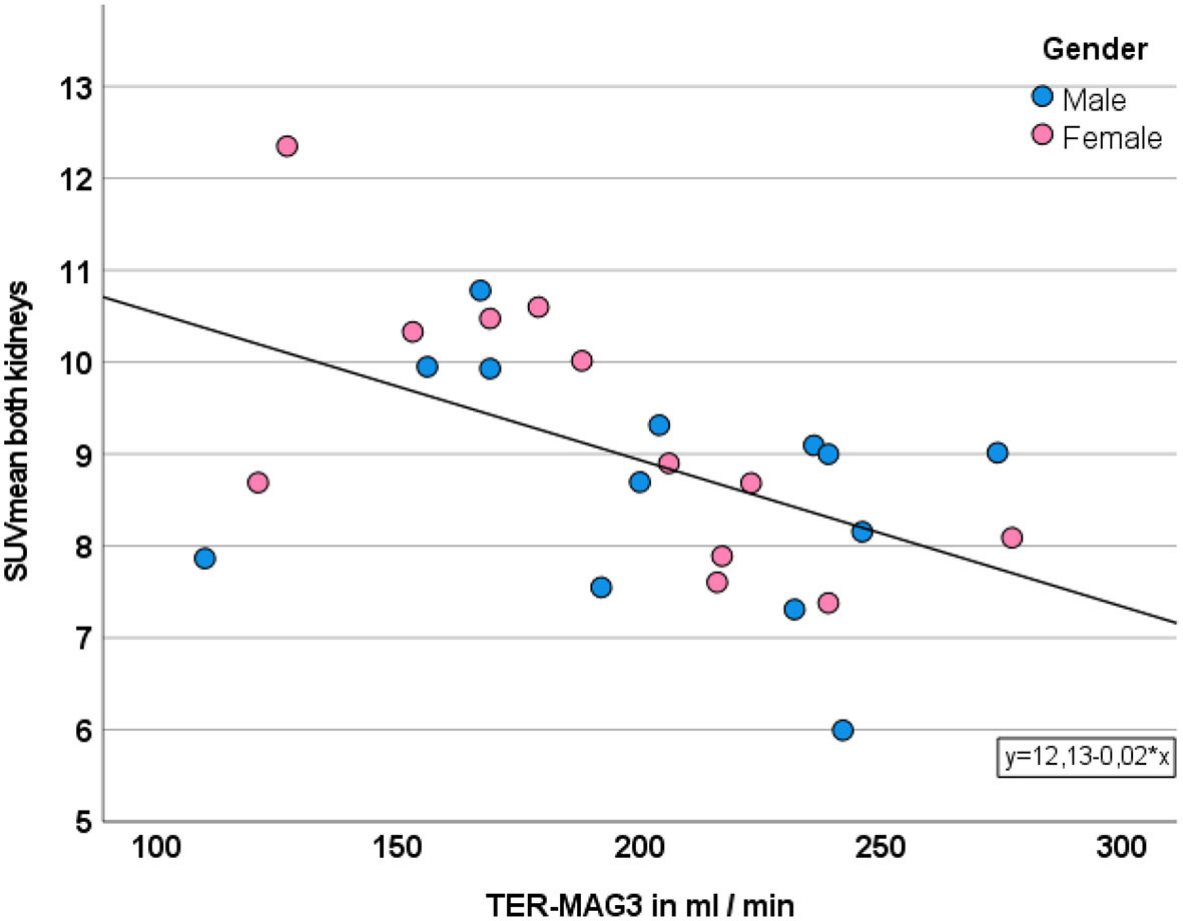
Correlation between SUVmean and TER. Gender is displayed in different colors.

Furthermore, the magnitude of SUVmean of the segmented renal parenchyma was moderately and negatively correlated with TER-MAG (r: −0.519, *p* < 0.001) and GFR_CDK − EPI_ (r: −0.555, *p* < 0.001) as shown in Figure 3.

SUVmean of the blood pool at 32 ± 0.5 min p.i. correlated negatively and moderately with TER-MAG (*r*: −0.590 *p* = 0.002) and GFR_CDK − EPI_ (*r* = −0.438, *p* = 0.028).

In contrast, total kidney accumulation (TKA_5 − 15_) as well as the volume of [^68^Ga]Ga-ha DOTATATE avid renal parenchyma showed no significant correlation with TER-MAG (*p* = 0.241 and *p* = 0.601) or GFR_CDK − EPI_ (*p* = 0.135 and *p* = 0.713).

### 3.4. PET-based assessment of split renal function

**Figure 4:**
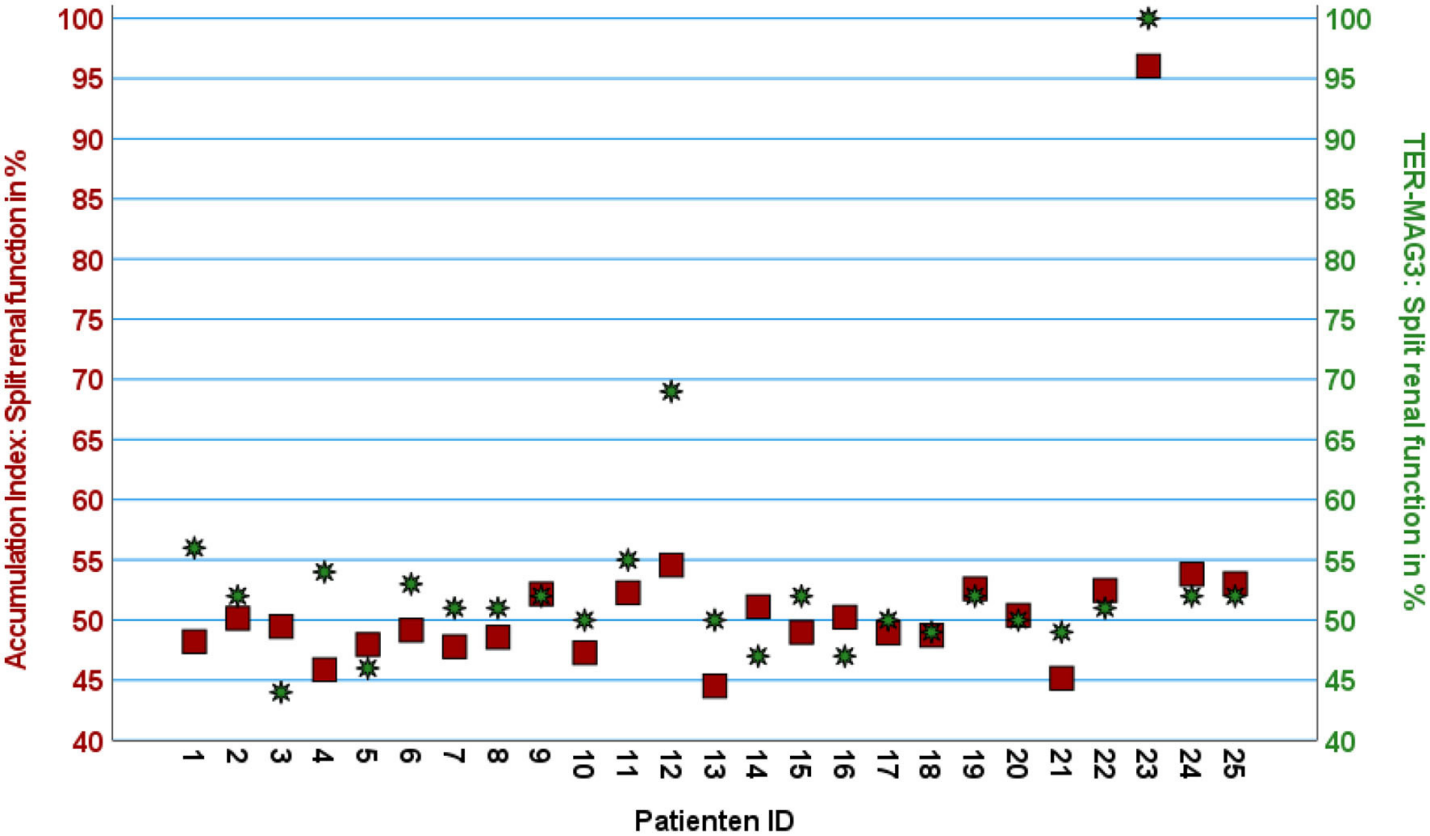
Scatter plot with two Z-axes displaying split renal function calculated using ACI on the left axis (red squares) and TER-MAG on the right axis (green stars) given for each patient in percent of the left kidney.

**Figure 5:**
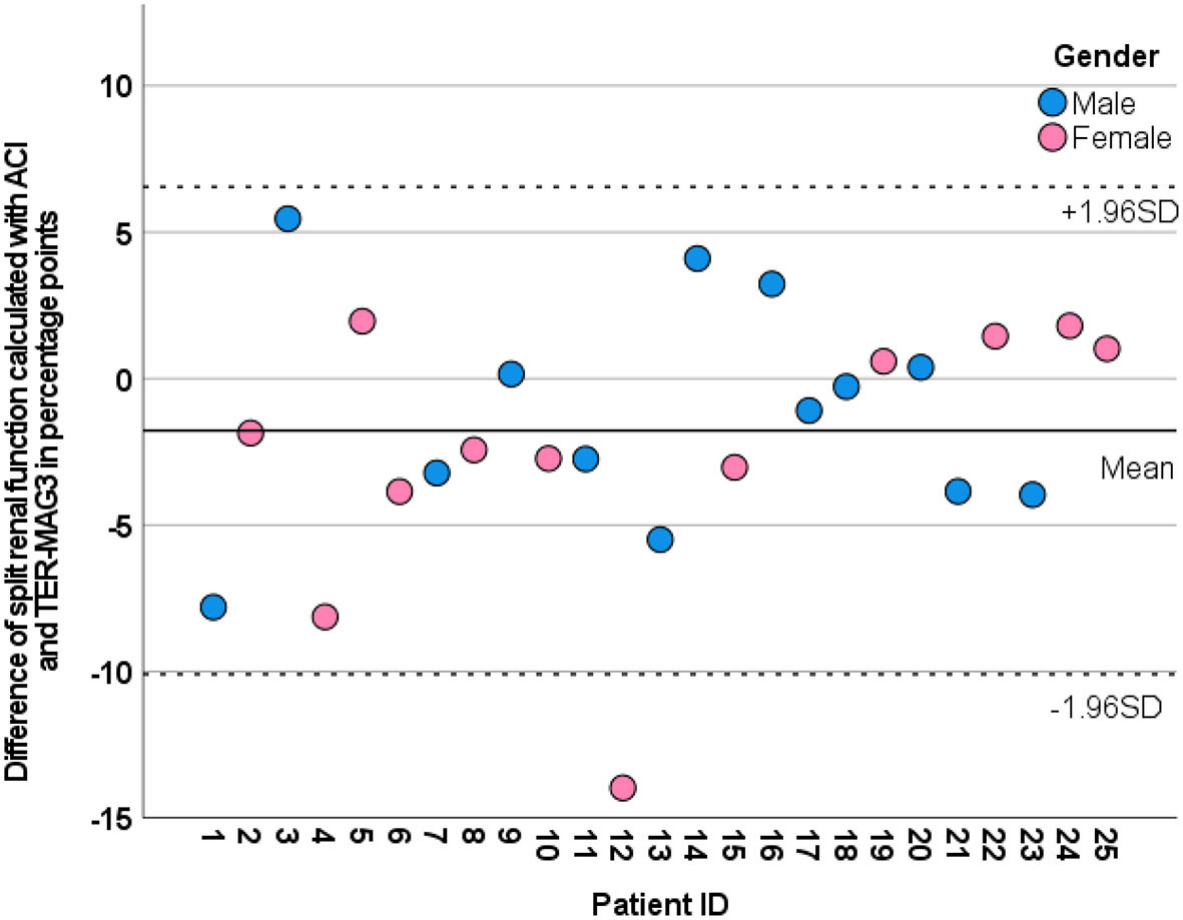
Modified Bland–Altman plot illustrating the deviation between the split renal function of the left kidney calculated by ACI (dots) from the reverence method MAG3 scintigraphy (0-line) for each patient. Gender is color coded. The mean value and ±1.96 SD are plotted as a line and dotted line.

Split renal function as estimated using ACI attained the highest correlation with renal scintigraphy, with a very strong correlation of *r* = 0.916 (*p* < 0.001). The mean difference between the split renal function determined by renal scintigraphy and ACI was 1.8 ± 4.2 % points. In 96% of patients (*n* = 24/25), the deviation between split renal function determined by ACI and renal scintigraphy was < 10 % points. There was one outlier (patient ID 12) with a difference of 14 % points as illustrated in Figures 4, 5.

Further estimation of split renal function with SUVmean (*r*: −0.740, *p* < 0.001), total kidney accumulation (TKA_5 − 15_) (*r*: 0.623, *p* < 0.001), or the volume of the [^68^Ga]Ga-ha DOTATATE avid parenchymal volume (*r*: 0.905, *p* < 0.001) resulted in moderate to strong correlations with the gold standard renal scintigraphy. Blood pool corrected TKI_bpc_ did not improve correlation with renal scintigraphy (*r*: 0.479, *p* = 0.015).

### 3.5. PET-based assessment of absolute single-sided renal function

#### 3.5.1. Accumulation index

**Figure 6:**
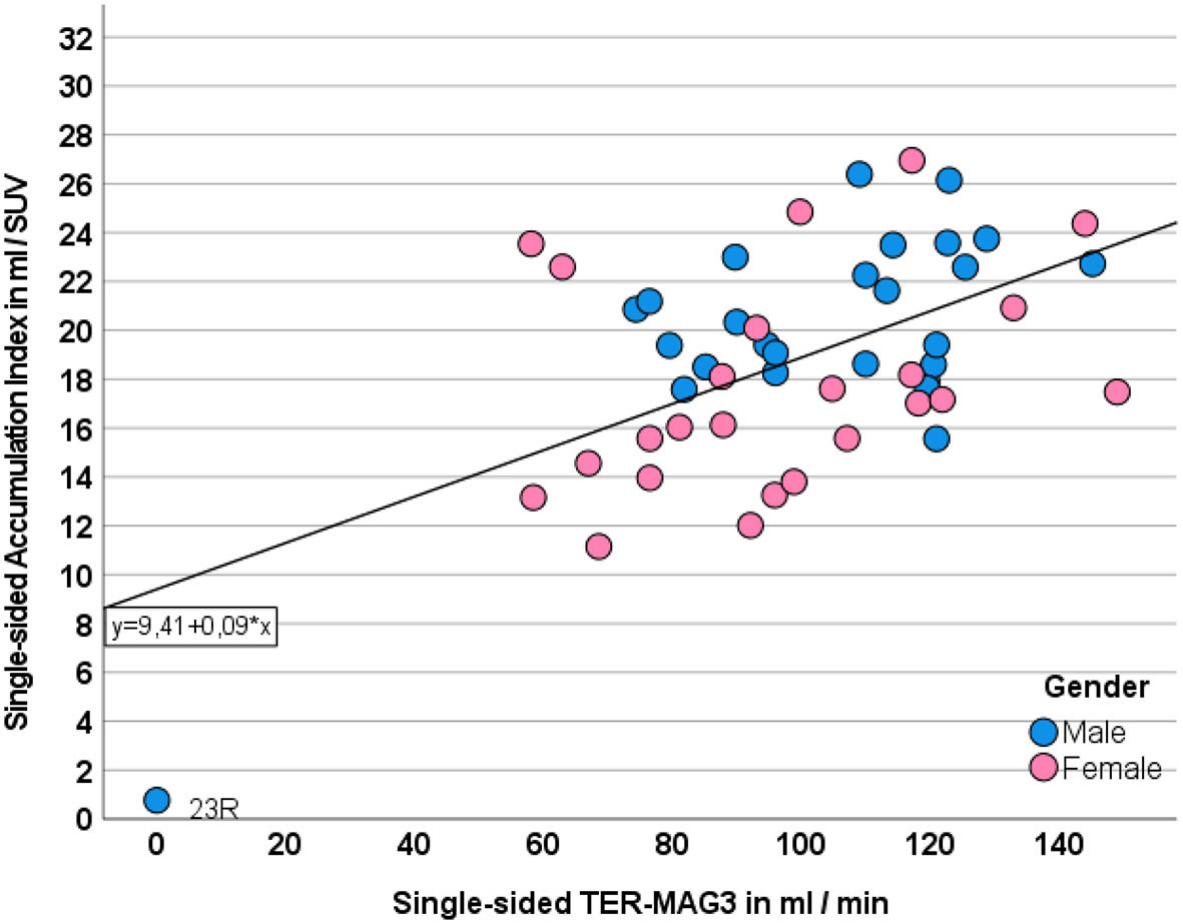
Correlation between single-sided ACI and TER-MAG. Gender is displayed in different colors.

The accumulation index correlated significantly with the absolute single-sided TER-MAG (r: 0.549, *p* < 0.001, *n* = 50) and the side-separated GFR_CDK − EPI_ values (0.549, *p* < 0.001 *n* = 50). In particular, a hydronephrosis grade IV kidney with severe parenchymal atrophy was identified as non-functional despite the presence of residual parenchyma (patient ID 12, kidney ID: 23 R, TKI 5 ml, TER-MAG 0 ml/min) as shown in Figure 6.

#### 3.5.2. SUVmean

**Figure 7:**
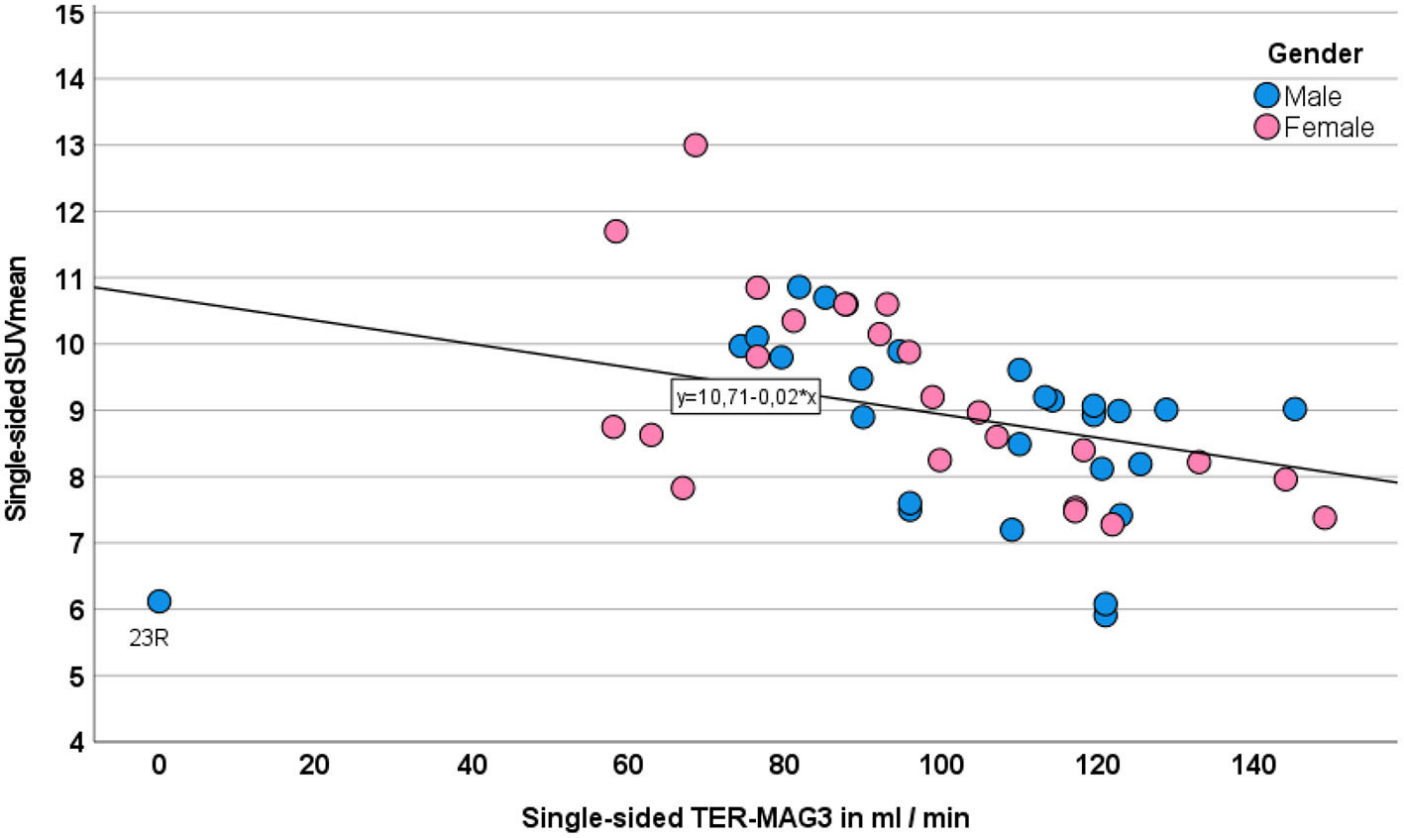
Correlation between single-sided SUVmean and TER-MAG. Gender is displayed in different colors.

SUVmean measurements of the renal parenchyma correlated negatively but only moderately with side-separated TER-MAG (*r*: −0.330, *p* = 0.019) and GFR_CDK − EPI_ (*r*: −0.337, *p* = 0.017) as shown in Figure 7. The non-functional, shrunken kidney in one patient had no relevant uptake, resulting in an extreme value that negatively affected the correlation as shown in the scatterplot Figure 7.

#### 3.5.3. Total Kidney Accumulation and parenchyma volume

TKA_5 − 15_ trended toward a non-significant, negative correlation with TER-MAG (*r*: −0.243, *p* = 0.241) and GFR_CKD − EPI_ (*r*: −0.308 *p* = 0.135).

The volume of the [^68^Ga]Ga-ha DOTATATE avid renal parenchymal showed a borderline insignificant correlation with TER-MAG and GFR_CDK − EPI_ (*r*: 0.272 and *r*: 0.272, *p* = 0.056).

Table 2 summarizes different correlations.

**Table 2:** Correlation between SSR-PET-based renal function estimation and reference TER-MAG3 scintigraphy.

	**Accumulation index (ACI)**	**SUVmean**	**Total kidney accumulation (TKA_5 − 15_)**	**[^68^Ga]Ga-ha DOTATATE avid renal parenchymal volume**
**Single-sided renal function**				
Split renal scintigraphy (MAG3)	**0.916** ^**^	**0.740** ^**^	**0.623** ^**^	**0.905** ^**^
Absolute single-sided renal function (Bubeck's method)	**0.549** ^**^	–0.330^*^	0.056	0.272
**Absolute renal function**				
Tubular extraction rate (MAG3)	**0.482** ^*^	**0.519** ^**^	0.243	0.110
GFR	**0.461** ^*^	–0.555^**^	−0.308	0.272

* *p* < 0.05,

** *p* < 0.01.

Significant results are highlighted in bold.

### 3.6. Tumor sink effect

The average MTV was 4,376 ± 6,215 ml x SUV. The mean %ID was 6.3 ± 9.0% with a range of 0.1–27.2%. The MTV showed a moderate, negative correlation with the blood pool SUVmean (*r*: −0.433, *p* = 0.031). The %ID correlated weakly negatively with the SUVmean of renal parenchyma (*r*: −0.403, *p* = 0.046) and moderately negatively with SUVmean blood pool (*r*: −0.416, *p* = 0.038). No correlation was observed between MTV or %ID and renal function parameters (ACI, GFR, and TER-MAG 3).

## 4. Discussion

To our knowledge, this is the first study that demonstrates that split renal function, as well as absolute renal function, can be estimated from routine static oncologic SSR-PET/CT with a ^68^Ga-labeled DOTA-conjugated peptide.

The data of this pilot study indicate that the amount of [^68^Ga]Ga-ha DOTATATE accumulating in the renal parenchyma correlates with the renal tubular and glomerular function and consequently with the split renal function. This might be of high clinical relevance, as patients with inferior renal function reveal a significantly higher renal absorbed dose and higher grade of hematological toxicity during treatment with ^177^Lu-DOTATATE (10).

Renal uptake of DOTA-conjugated peptides is reported to be predominantly caused by renal reabsorption in the proximal tubules, especially the megalin receptors (3, 17). A biochemical explanation might be an increased DOTATATE reabsorption in the megalin receptor or higher megalin transporter density, although the exact mechanism is still unclear (3). In addition, slower blood pool clearance could increase tracer bioavailability and thus renal DOTATATE retention (3, 10). This hypothesis is based on the ^68^Ga-DOTA-conjugated peptide kinetics with an early peak at about 5 min in the renal parenchymal and an early glomeruli washout as well as blood pool clearance in patients with unrestricted renal function (3, 18, 19). Of particular note, somatostatin receptors have been shown to have no significant effect on renal DOTATATE uptake (3).

Previous attempts of SSR-PET-based renal function estimation might have failed due to the later acquisition time of 60–120 min p.i. and an incomplete parenchyma segmentation (20, 21). According to the literature data, the parenchymal activity remains relatively constant at a low level after 30 min p.i., owing to tubular reabsorption (3, 17, 18).

As another possible confounding factor for the SSR-PET-based assessment of renal function, tumor sequestration of somatostatin analogs has to be discussed, which is the main cause of the tumor sink effect (22). This effect was also evident in our cohort with a decrease in tracer concentration in the renal parenchyma and the blood pool in patients with high tumor masses. However, this did not significantly affect the accuracy of renal function determination by ACI.

The SUVmean of the segmented renal parenchyma correlated with the gold standard renal scintigraphy for both split function and absolute renal function. However, the SUVmean-based measurements of absolute split function revealed an outlier, which was attributed to grade IV hydronephrosis with severe atrophy and only little residual parenchyma. In this particular case, residual renal parenchyma was within the range of healthy renal tissue with an SUV of 6 and higher than blood pool or muscle tissue. This semiquantitatively estimated tracer uptake may be due to the smallest amounts of residual functional renal parenchyma (parenchyma volume 3 ml), which can be underestimated in scintigraphy due to the partial volume effect. Beyond that, a tracer fixation to the damaged renal tubule independent of the renal function might be considered.

Regardless of the underlying effect, a major risk of the SUV method is to overestimate the partial function of a small kidney, by neglecting kidney volume and thus the total renal corpuscular volume (total number of glomeruli x mean renal corpuscular volume) (23).

An approach to solving this issue is to implement the volume of SSR-avid renal parenchymal, which correlated significantly with split renal function. The volume of SSR-avid renal parenchymal seems to be a significant indicator in addition to SUV as a higher number of functioning nephrons can be assumed in a larger parenchyma volume, thus increasing the filtration rate. Furthermore, the pharmacokinetic data indicate that the DOTATATE concentration of ~30 min p.i. is predominantly attributed to the residual activity in the proximal tubules, and only a relatively small part of the parenchymal activity should be attributed to the tubular reabsorption at this early time point (3, 17, 18).

Based on the negative correlation of the parenchymal SUV and the presumed positive correlation of the tracer filtering renal parenchymal volume with TER, the simple isocontour-based ACI_5 − 15_ was defined by dividing the parenchyma volume by the SUV.

This ACI represented a highly significant predictor of split renal function and single-sided absolute function. A case of the non-functional kidney was identified due to the small volume of tracer-avid parenchyma.

From a clinical point of view, the ACI achieved the highest correlation with renal scintigraphy as the reference standard with an acceptable deviation of <5 % points. However, in one patient, the deviation of split renal function calculated with ACI (left 55: right 45%) and MAG3 scintigraphy (left 69%: right 31%) was more than 10 % points before PRRT. Though the unbalanced split renal function was confirmed in the 2-month follow-up scintigraphy (after the first PRRT cycle), the partial function of the dominant kidney later declined under second–fourth PRRT cycles to 50:50%. Furthermore, TER-MAG declined from 216 ml/min/1.73 m^2^ to 165 ml/min/1.73 m^2^ (lower limit of the norm: 192 ml/min/1.73 m^2^). In addition to variances in the measurement method itself, differences in the measured structure can lead to different results. Minor glomerular damages may remain occult in the tubular excretion of MAG3 tracers, whereas glomerular-filtered tracers like [^68^Ga]Ga-ha DOTATATE may be more sensitive.

In recent years, great efforts have been made to estimate the split renal function without radiation exposure. A processing method is functional MRI (24, 25). Apart from the time and financial expense of an additional examination, gadolinium-based contrast media must still be used for accurate quantification (26). Unfortunately, the risk for side effects of gadolinium-based contrast agents, such as nephrogenic systemic fibrosis (NSF), increases with decreasing renal function; however, these are the individuals who require renal function measurements the most (27). In addition, older adults, in particular, may not qualify for MRI due to claustrophobia, ferromagnetic material, or pacemakers. Here, the integration of split renal function estimation into follow-up examinations seems to be more appropriate.

### 4.1. Limitations

First, renal scintigraphy as the standard of reference has some limitations. In particular, the [^99m^Tc]Tc-MAG3 tracer used for renal scintigraphy is tubularly extracted, whereas SSR-targeting peptide tracers are mainly subject to glomerular filtration and partially reabsorbed in the proximal tubule via the Megalin receptor (3, 17). Furthermore, owing to the lower spatial resolution and two-dimensional imaging, planar scintigraphy tends to systematically underestimate small parenchymal volumes. However, renal scintigraphy provides an accurate and robust assessment of side-separated renal function and remains the gold standard in the majority of clinical trials.

One alternative to [^99m^Tc]Tc-MAG3 undergoing predominantly tubular excretion would be the use of [^99m^Tc]Tc -diethylenetriaminepetaacetic acid [^99m^Tc]Tc-DTPA) which is subject to glomerular filtration. [^99m^Tc]Tc-DTPA or ^68^Ga-labeled EDTA would potentially have been more comparable with the complex filtration of the [^68^Ga]Ga-ha DOTATATE tracer but is not routinely used for renal function monitoring (4).

Furthermore, owing to the retrospective study design, the period between PET and scintigraphy was not predefined and was 16 days on average. Additionally, the calculation of GFR may show uncertainties and is dependent on the hydration status at the time of blood sampling, which remains unknown in this retrospective study (14).

However, as TER-MAG and GFR are highly correlated in determining overall renal function, TER-MAG may be used as a sufficient surrogate marker for the estimation of glomerular filtration rate.

Optimization of the ACI with the weighting of the individual factors and implementation of the blood pool as a tracer clearance indicator should be performed on a larger patient population to establish a more accurate SSR-PET-based renal function assessment.

## 5. Conclusion

The data from this pilot study indicate that absolute single-sided renal function can be estimated using SSR-PET with DOTA-conjugated peptide tracers such as [68 Ga]Ga-ha DOTATATE acquired prior to PRRT. It was shown that the remaining parenchymal tracer uptake and blood pool activity at about 30 min p.i. correlate negatively with the tubular extraction rate and glomerular filtration rate. The proposed “**Ac**cumulation **I**ndex” revealed a highly significant correlation with the absolute TER-MAG and GFR as well as split renal function and absolute single-sided renal function. Further validation of these results in a larger cohort is needed.

## Data availability statement

The raw data supporting the conclusions of this article will be made available by the authors, upon request, subject to national data protection regulation.

## Ethics statement

The studies involving human participants were reviewed and approved by Ethik-Kommission an der Medizinischen Fakultät der Eberhard-Karls-Universität und am Universitätsklinikum Tübingen. The patients/participants provided their written informed consent to participate in this study.

## Author contributions

MW: visualization, writing—original draft, data curation, investigation, formal analysis, conceptualization, and methodology. KS: data curation and investigation. SU: visualization. SC-V: software and formal analysis. SB: methodology. JV, MW, and PG: validation. KN and CF: funding acquisition and resources. CF, HD, FS, and SU: writing—review and editing. HD: project administration, supervision, conceptualization, and methodology. KS: writing—original draft, formal analysis, and methodology. All authors contributed to the article and approved the submitted version.
